# Respiratory SARS-CoV-2 Infection Causes Skeletal Muscle Atrophy and Long-Lasting Energy Metabolism Suppression

**DOI:** 10.3390/biomedicines12071443

**Published:** 2024-06-28

**Authors:** Sachiko T. Homma, Xingyu Wang, Justin J. Frere, Adam C. Gower, Jingsong Zhou, Jean K. Lim, Benjamin R. tenOever, Lan Zhou

**Affiliations:** 1 Department of Neurology, Boston University Chobanian & Avedisian School of Medicine, Boston, MA 02118, USA; 2 Department of Neurology, Hospital for Special Surgery, New York, NY 10021, USA; 3 Department of Microbiology, Icahn School of Medicine at Mount Sinai, New York, NY 10029, USA; 4 Clinical and Translational Science Institute, Boston University Chobanian & Avedisian School of Medicine, Boston, MA 02118, USA; 5 College of Nursing and Health Innovation, University of Texas at Arlington, Arlington, TX 76010, USA; 6 Department of Microbiology, Grossman School of Medicine, New York University, New York, NY 10016, USA

**Keywords:** COVID-19, long COVID, influenza, muscle fatigue, muscle atrophy, energy metabolism, mitochondria, interferons, tumor necrosis factor-alpha

## Abstract

Muscle fatigue represents the most prevalent symptom of long-term COVID, with elusive pathogenic mechanisms. We performed a longitudinal study to characterize histopathological and transcriptional changes in skeletal muscle in a hamster model of respiratory SARS-CoV-2 infection and compared them with influenza A virus (IAV) and mock infections. Histopathological and bulk RNA sequencing analyses of leg muscles derived from infected animals at days 3, 30, and 60 post-infection showed no direct viral invasion but myofiber atrophy in the SARS-CoV-2 group, which was accompanied by persistent downregulation of the genes related to myofibers, ribosomal proteins, fatty acid β-oxidation, tricarboxylic acid cycle, and mitochondrial oxidative phosphorylation complexes. While both SARS-CoV-2 and IAV infections induced acute and transient type I and II interferon responses in muscle, only the SARS-CoV-2 infection upregulated TNF-α/NF-κB but not IL-6 signaling in muscle. Treatment of C2C12 myotubes, a skeletal muscle cell line, with combined IFN-γ and TNF-α but not with IFN-γ or TNF-α alone markedly impaired mitochondrial function. We conclude that a respiratory SARS-CoV-2 infection can cause myofiber atrophy and persistent energy metabolism suppression without direct viral invasion. The effects may be induced by the combined systemic interferon and TNF-α responses at the acute phase and may contribute to post-COVID-19 persistent muscle fatigue.

## 1. Introduction

Severe acute respiratory syndrome coronavirus 2 (SARS-CoV-2) has caused a global pandemic of coronavirus disease 2019 (COVID-19) since March 2020. Although the situation has greatly improved, thanks to the development of vaccines, advances in the treatment of acute infections, and the evolution of less virulent strains, many new COVID-19 cases and related morbidity and mortality are encountered every day worldwide. COVID-19 impacts human health beyond acute infection. COVID-19 long-haul symptoms are relatively prevalent across different age groups [1,2], and managing these symptoms has become a challenge. The condition in which the symptoms persist beyond 12 weeks after an acute viral infection with no alternative diagnosis has been defined as post-COVID-19 syndrome [3].

Post-COVID-19 syndrome can manifest many symptoms, the majority of which are neurological and neuropsychiatric, including myalgia, fatigue, brain fog, headaches, insomnia, and anxiety, among others [4,5]. Muscle fatigue represents the most prevalent symptom, as revealed by several large cohort studies, and it can occur regardless of the severity of the initial viral infection [5,6,7,8,9]. Although myalgia and fatigue are common with acute respiratory viral infections such as influenza, the symptoms are often more severe and long-lasting when associated with SARS-CoV-2 [10], implicating prolonged structural and functional abnormalities in skeletal muscle after acute respiratory SARS-CoV-2 infection. There are several published histopathological examinations of the skeletal muscle of patients who suffered from long COVID-19 [11,12,13,14]. These studies showed a variety of pathological changes, including muscle atrophy, inflammation, mitochondrial abnormalities, and capillary injury. Recent studies have also shown reduced mitochondrial oxidative capacity in patients with long COVID [12,13,14].

To better understand the molecular mechanisms underlying the development and persistence of myalgia and fatigue associated with COVID-19, we performed a longitudinal study to characterize the histopathological and transcriptional responses of skeletal muscle to respiratory SARS-CoV-2 infection and benchmarked the findings to influenza A virus (IAV) infection, utilizing the golden hamster as a model system. The hamster model has been proven to largely phenocopy COVID-19 biology, and it displays severe lung morphology and a tropism that matches what is observed in human patients [15,16,17]. Our study showed no direct viral invasion but myofiber atrophy, which was accompanied by persistent suppression of the genes related to myofibers, ribosomal proteins, and mitochondrial oxidative metabolism in the SARS-CoV-2 group. It downregulated both cytoplasmic and mitochondrial ribosome protein genes, likely impairing protein synthesis. It also downregulated many nuclear genes, but not mitochondrial genes, involved in fatty acid β-oxidation, the tricarboxylic acid (TCA) cycle, and all five oxidative phosphorylation (OXPHOS) complexes. In contrast, no myofiber atrophy or persistent gene expression changes were observed in the IAV-infected hamsters. In addition to the transient type I and type II interferon responses at the acute phase of either infection, only the SARS-CoV-2 infection induced TNF-α but not IL-6 response in skeletal muscle. In vitro co-treatment of differentiated C2C12 cells, a skeletal muscle cell line, with IFN-γ and TNF-α greatly impaired mitochondrial respiration and shifted energy metabolism from mitochondrial oxidative respiration to glycolysis. Our findings suggest that the combined systemic interferon and TNF-α responses during acute respiratory SARS-CoV-2 infection might induce a long-lasting suppression of mitochondrial oxidative energy metabolism and myofiber atrophy, causing acute and persistent muscle symptoms.

## 2. Materials and Methods

### 2.1. Golden Hamster Models

Six-week-old male Golden Syrian hamsters (*Mesocricetus auratus*) were obtained from Charles River Laboratories (Wilmington, MA, USA). Hamsters were acclimated to the CDC/USDA-approved Biosafety Level 3 (BSL-3) facility at the Center for Comparative Medicine and Surgery at the Icahn School of Medicine at Mount Sinai (New York, NY, USA). When the hamsters reached 10 weeks of age, they were randomly divided into nine groups for induction of three different kind of infections and for evaluation at three time points post-infection (three hamsters/infection/time point). Three infection groups include (1) mock-infected group: intranasally treated with PBS; (2) SARS-CoV-2-infected group: intranasally infected with 1000 pfu (total volume 100 μL) of SARS-CoV-2 (USA-WA1/2020); and (3) IAV-infected group: intranasally infected with 100,000 pfu (total volume 100 μL) of H1N1 IAV (A/California/04/2009). Intranasal administration was performed under ketamine/xylazine anesthesia. Hamsters were housed for 3-, 30-, and 60 days post-infection (dpi) before being euthanized via sodium pentobarbital and intracardiac perfusion with PBS. After perfusion, quadriceps muscle was exposed and harvested. Half of each sample was placed into a 4% paraformaldehyde (PFA) solution to be fixed for 48 h before being transferred to PBS. The other half was placed into “lysing matrix A” homogenization tubes (MP Biomedicals, Santa Ana, CA, USA) filled with TRIzol (Thermo Fisher Scientific, Waltham, MA, USA) and homogenized before being frozen at −80 °C.

### 2.2. Hematoxylin and Eosin (H&E) Staining

Paraffin-embedded tissue blocks were cut into 6-μm sections and mounted on charged glass slides (Thermo Fisher Scientific). Sections were deparaffinized by immersion in xylene and rehydrated in decreasing ethanol dilutions. Slides were then stained using the Hematoxylin and Eosin stain kit (Vector Laboratories, Newark, CA, USA) following the manufacturer’s instructions. Slides were dehydrated by immersion in increasing concentrations of ethanol, cleared with xylene, and coverslipped. Standard microscope images were acquired using a Nikon E800 microscope with spot camera and software version 5.0 (Diagnostic Instruments Inc., Sterling Heights, MI, USA).

### 2.3. Immunohistochemistry (IHC)

Paraffin sections were used for immunostaining. Antigen retrieval was performed for 30 min in a pressure cooker with slides immersed in antigen retrieval buffer (Tris/EDTA pH 9.0). Tissue sections were blocked in Tris-Buffered Saline (TBS) with 3% bovine serum albumin and 5% normal goat serum (Vector laboratories) or 5% normal rabbit serum (Vector laboratories) for 2 h at room temperature. For brightfield IHC, the primary antibody of SARS nucleocapsid protein (NP100-56576, Novus Biologicals, Centennial, CO, USA) was added to slides at a 1:250 dilution. Sections were incubated overnight at 4 °C. Slides were washed in TBS with 0.025% Triton X-100 prior to immersion in 0.3% hydrogen peroxide in TBS for 15 min. Slides were washed again, and a horseradish peroxidase-linked secondary antibody system (PK-6100, Vector Laboratories) and diaminobenzidine substrate were used for the detection of immunoreactive signals according to the manufacturer’s instructions. Slides were then dehydrated by immersion in increasing concentrations of ethanol, cleared with xylene, and coverslipped. For immunofluorescent microcopy, the sections were incubated overnight at 4 °C with primary antibodies of SARS-CoV-2 N protein (GXT635679, Genetex, Irvine, CA, USA) and SARS-CoV-2 S protein (ZMS1076, Sigma-Aldrich, St. Louis, MO, USA) at a 1:1000 dilution. The sections were then washed and incubated with secondary antibodies conjugated with Alexa Fluor 568 for the N protein and Alexa Fluor 647 for the S protein (A-11011 and A-21235, respectively, Thermo Fisher Scientific) at a 1:1000 dilution for 1 h. The slides were coverslipped after 4′,6-diamidino-2-phenylindole (DAPI) staining of nuclei. Fluorescent microscope images were acquired using a Zeiss Axio Observer microscope and Zen 2.6 (Blue edition) software (Carl Zeiss, White Plains, NY, USA). Autofluorescence was captured by the green fluorescence channel to monitor false-positive cells for SARS-CoV-2 proteins.

### 2.4. Muscle Fiber Type Composition

After IHC using primary antibodies against slow skeletal myosin heavy chain (ab11083, Abcam, Waltham, MA, USA, 1:200 dilution) and fast skeletal myosin heavy chain (ab51263, Abcam, 1:400 dilution), quantification of the number of each type of myofiber and myofiber cross-sectional area were performed. The fiber type distribution was expressed as a percentage of slow or fast myosin heavy chain expressing fibers in the total fibers on the sections.

### 2.5. Transmission Electron Microscopy (TEM)

Tissues for TEM were processed and imaged in the Boston University School of Medicine TEM core facility. Tissue blocks of quadriceps were post-fixed with 3% glutaraldehyde solution (Electron Microscopy Sciences, Hatfield, PA, USA) and 3% paraformaldehyde (Electron Microscopy Sciences) in 0.1 M phosphate buffer (PB, pH 7.4) for 24 h at 4 °C. After rinsing in 0.1 M PBS, tissue blocks were fixed with 1% osmium tetroxide (Electron Microscopy Sciences) for 1.5 h, rinsed in water, and then dehydrated in a series of increasing acetone concentrations (50%, 90%, and 100%). The samples were then infiltrated and block-embedded in epoxy resin (Electron Microscopy Sciences). Ultrathin sections (50 nm) were prepared with an ultra-microtome (Leica, Wetzlar, Germany) and collected into copper mesh grids (Ted Pella, Redding, CA, USA). Grids were counterstained with 4% uranyl acetate (Electron Microscopy Sciences) for 5 min at 60 °C, followed by rinses in filtered distilled water and then in 0.4% lead citrate (Ted Pella) for 45 s at room temperature. The sections were imaged and photographed using a JEM1400 electron microscope (JEOL) connected to an AMT NanoSprint-43M-B Mid-Mount CMOS camera (AMT701, Advanced Microscopy Techniques Corp., Woburn, MA, USA). Individual subsarcolemmal (SS) and intermyofibrillar (IMF) mitochondria from three hamsters of each group were manually traced in longitudinal orientation using ImageJ (NIH) and quantified using the morphological and shape descriptors including area (μm^2^), perimeter (μm), Feret’s diameter (longest distance between any two points within a given mitochondrion, μm), and aspect ratio (major axis divided by minor axis, which is a measure of the length to width ratio).

### 2.6. Quantitative Reverse-Transcription Polymerase Chain Reaction (qRT-PCR)

Total RNA was isolated from homogenized muscle samples by TRIzol (Thermo Fisher Scientific) and was further cleaned and treated with DNase using Directzol RNA mini-prep (Zymo Research, Irvine, CA, USA). One microgram of total RNA from each tissue was reverse-transcribed into cDNA with oligo dT primers using SuperScript IV reverse transcriptase (Thermo Fisher Scientific). Quantitative polymerase chain reaction (qPCR) was performed using primers described in Supplementary Table S1, PowerTrack SYBR Green Master Mix (Applied Biosystems, Waltham, MA, USA), and Quantstudio 12K Flex qPCR system (Applied Biosystems).

### 2.7. Quantification of Mitochondrial DNA (mtDNA) Content by Real-Time PCR

Total DNA was isolated from paraffin sections of quadriceps using Quick-DNA FFPE MiniPrep (Zymo Research). MtDNA content was gauged by the mtDNA/nuclear DNA (nDNA) ratio measured by qPCR of NADH-ubiquinone oxidoreductase chain 1 gene (*nd1*) and beta-actin gene (*actb*) using primers described in Supplementary Table S1 [18].

### 2.8. Bulk RNA Sequencing (RNAseq)

One hundred nanograms of total RNA from each sample was enriched for polyadenylated RNA and prepared for next-generation sequencing using NEBNext Ultra II Directional RNA Library Prep Kit (New England Biolabs, Ipswich, MA, USA) and following the manufacturer’s instructions. Prepared libraries were sequenced on an Illumina NextSeq 2000 platform. Prepared libraries were sequenced on an Illumina NextSeq 2000 instrument. FASTQ files were aligned to hamster genome build MesAur1.0 using STAR [19] (version 2.7.9a). Ensembl-Gene-level counts were generated using featureCounts (Subread package, version 1.6.2) and Ensembl annotation build 105 (uniquely aligned proper pairs, same strand). FASTQ quality was assessed using FastQC (version 0.11.7) and alignment quality was assessed using RSeQC (version 3.0.0). Differential expression was assessed using Wald test implemented in DESeq2 R package (version 1.32.10). Correction for multiple hypothesis testing was accomplished using Benjamini-Hochberg false discovery rate (FDR). Human homologs of hamster genes were identified using NCBI ‘gene orthologs’ table (retrieved 28 March 2022). All analyses were performed using R environment for statistical computing (version 4.1.2). Gene Set Enrichment Analysis (GSEA) (version 2.2.1) [20] was used to identify biological terms, pathways, and processes that are coordinately up- or down-regulated within each pairwise comparison. Entrez Gene identifiers of human homologs of all genes in Ensembl Gene annotation were ranked by Wald statistic computed for each pairwise comparison. Ensembl Genes matching multiple hamster Entrez Gene identifiers and hamster genes with multiple human homologs (or vice versa) were excluded prior to ranking, so that the ranked list represents only those human Entrez Gene IDs that match exactly one hamster Ensembl Gene. Each ranked list was then used to perform pre-ranked GSEA analyses (default parameters with random seed 1234) using the Entrez Gene versions of the H (Hallmark), C2 CP (Biocarta, KEGG, PID, Reactome, WikiPathways), C3 (transcription factor and microRNA motif), and C5 (Gene Ontology, GO) gene sets obtained from Molecular Signatures Database (MSigDB), version 7.5.1.

### 2.9. Cell Culture and Cytokine Treatments

C2C12 mouse myoblasts (CRL-1772) from ATCC (Manassas, VA, USA) were grown in 0.1% gelatin-coated dishes in Dulbecco’s modified Eagle’s medium (Fisher Scientific) containing 4500 mg/L glucose and supplemented with 10% fetal bovine serum (Thermo Fisher Scientific) and 100 U/mL penicillin/streptomycin (Thermo Fisher Scientific) in a humidified 5% CO_2_ incubator at 37 °C. To induce differentiation, proliferating cultures that were near confluence were switched into a low-serum differentiation medium consisting of 2% horse serum (Thermo Fisher Scientific). Cytokine treatments were carried out with differentiating cells that had been in differentiation medium for 3 days after reaching confluence. Differentiating cultures were incubated for 24 and 48 h with mouse TNF-α (410-MT, R&D systems, Minneapolis, MN, USA, 10 ng/mL), universal type I interferon IFN-α (11200, PBL assay science, Piscataway, NJ, USA, 100U), mouse IFN-γ (485-MI, R&D systems, 100U), and mouse IL-6 (406-ML, R&D systems, 10 ng/mL) alone or in combination.

### 2.10. Immunoblotting

C2C12 myogenic cells were homogenized in RIPA buffer (50 mmol/L Tris-HCl pH 7.4, 150 mmol/L NaCl, 1% NP-40, 0.5% sodium deoxycholic acid, and 0.1% SDS) with protease inhibitors (Thermo Fisher Scientific), sonicated, and then centrifuged at 15,000× *g* for 15 min. The protein in the supernatant was quantified by a BCA protein assay (Thermo Fisher Scientific). Thirty micrograms of protein were subjected to SDS-PAGE under reducing conditions and transferred to polyvinylidene difluoride (PVDF) membranes (MilliporeSigma, Burlington, MA, USA). Blots were blocked in 3% non-fat skim milk or 3% BSA and washed three times with TBS containing 0.1% Tween. Membranes were incubated with primary antibodies against total OXPHOS cocktail (ab110413, Abcam, 1:250), COX17 (11464-1AP, Proteintech, Rosemont, IL, USA, 1:500), RPS3 (66046-1-lg, Proteintech, 1:500), RPL23 (A305-010A, Bethyl labolatories, Montgomery, TX, USA, 1:1000), or tubulin (2148S, Cell Signaling Technology, Danvers, MA, USA, 1:1000) and then washed. Signals were detected with IRDye 800- or 680-conjugated secondary antibodies (926-68020, 925-68021, LI-COR Biosciences, Lincoln, NE, USA, 1:5000) with appropriate species specificity. Immunoblots were visualized by Image Studio software version 2.1.10 (LI-COR Biosciences) that accompanies the LI-COR Odyssey infrared system (LI-COR Biosciences).

### 2.11. Bioenergetic Analysis

The oxygen consumption rate (OCR), extracellular acidification rate (ECAR), and ATP production rate of C2C12 cells were determined using an Agilent Seahorse XFe96 Analyzer following the manufacturer’s instructions. XF base medium, XFe96 culture plates, and the XF96 Extracellular Flux assay kit were purchased from Agilent Technologies (Santa Clara, CA, USA). C2C12 cells were grown and differentiated on 0.1% gelatin-coated XFe96 culture plates. On the third day in the differentiation medium, cells were treated with cytokine(s) for 24 h, as described above. After the treatment, cells were washed and switched to XF base medium supplemented with 10 mM glucose, 1 mM sodium pyruvate, and 2 mM glutamine and cultured in a CO_2_-free incubator at 37 °C for 1 h. OCR and ECAR were measured under basal conditions and after injections of a final concentration of 1 μM oligomycin, 0.5 μM FCCP, and/or 1 μM antimycin A combined with 1 μM rotenone. Following completion of the measurements, cells were washed with PBS and then lysed by adding 25 μL of RIPA lysis buffer to each well. The protein amount of the cell lysate in each well was determined by BCA assay. The OCR and ATP production rates were normalized to the protein amounts.

### 2.12. Statistics

All non-RNAseq statistical analyses, bar graphs, x-y plots, violin plots, and heat maps were prepared using GraphPad Prism 9 as described in the figure legends. The significance of these analyses was determined utilizing statistical tests, including ANOVA with post-hoc analyses. Specific post-hoc analyses and statistical thresholds are described in the figure legends.

## 3. Results

### 3.1. There Is No Evidence of Direct SARS-CoV-2 Viral Invasion of Skeletal Muscle in the Hamster Model

**Figure 1:**
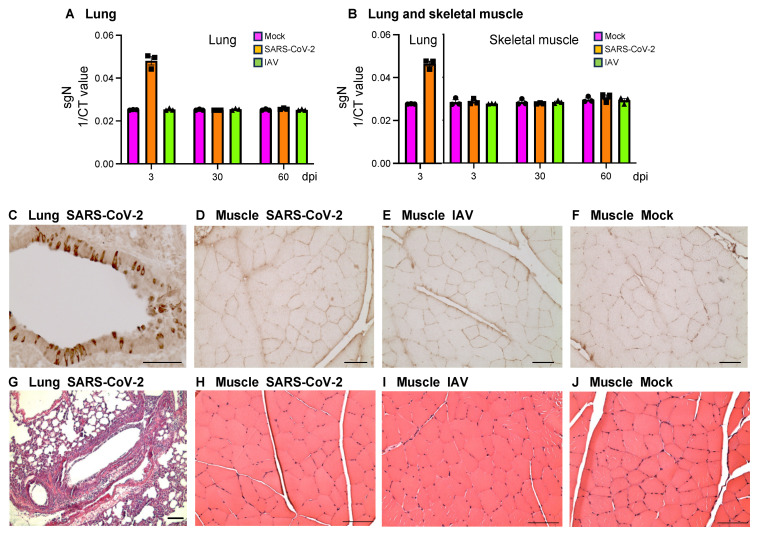
There is no evidence of direct SARS-CoV-2 invasion of skeletal muscle. (**A**,**B**) qRT-PCR of nucleoprotein subgenomic RNA (*sgN*) using RNA samples from lungs (**A**) and quadriceps muscles (**B**) of SARS-CoV-2-, influenza A virus (IAV)-, or mock-infected hamsters at 3-, 30-, and 60-days post-infection (dpi). *n* = 3 hamsters/infection group/time point. Each dot represents mean value of 3 replicates of each sample. Data are expressed as mean ± SEM. CT: cycle threshold. (**C**–**F**) Immunostaining of SARS-CoV-2 N protein using lung tissue from SARS-CoV-2-infected hamsters at 3 dpi (**C**) and quadriceps muscles from SARS-CoV-2- (**D**), IAV- (**E**), and mock- (**F**) infected hamsters at 3 dpi. (**G**–**J**) H&E staining of lung tissue from SARS-CoV-2-infected hamsters at 3 dpi (**G**) and quadriceps muscles from SARS-CoV-2- (**H**), IAV- (**I**), and mock- (**J**)-infected hamsters at 3 dpi. Images are representatives of 3 hamsters/infection group/time point (**C**–**J**). Bar = 50 μm (**C**–**F**). Bar = 100 μm (**G**–**J**).

SARS-CoV-2, IAV, or PBS were intranasally delivered to golden hamsters at 10 weeks of age. Inoculation dosages were determined based on the prior studies that achieved comparable kinetics and viral loads in the SARS-CoV-2 and IAV model systems [15,16,17]. Both respiratory RNA viruses replicated in the lungs of golden hamsters, with the viral levels reaching the highest at 3 dpi and then becoming undetectable by 7 dpi [16,17]. Based on the results, we assessed the SARS-CoV-2 virus at 3 dpi in quadriceps by RNAseq, qRT-PCR, IHC, and TEM. RNAseq analysis detected SARS-CoV-2 transcript of 934.7 ± 503.4 (mean ± SD) reads per million (RPM) in lungs but only 0.80 ± 0.82 RPM (*p* < 0.05) in muscle at 3 dpi that was most likely from blood contamination, as muscle is rich in blood vessels. The SARS-CoV-2 transcript was not detectable in muscle at 30 or 60 dpi. qRT-PCR detected a high level of SARS-CoV-2 nucleoprotein subgenomic (*sgN*) transcript in the lungs of SARS-CoV-2-infected animals at 3 dpi but not at 30 or 60 dpi or in mock- or IAV-infected animals at any time points (Figure 1A). In quadriceps muscle, no significant expression of *sgN* was detected in any infection groups at any time points (Figure 1B). Immunostaining of SARS-CoV-2 N protein showed robust signals in bronchial epithelial cells (Figure 1C), bronchioles, and alveoli (Supplementary Figure S1B,E) in SARS-CoV-2-infected hamsters at 3 dpi but not 30 dpi (Supplementary Figure S1C,F) or in mock-infected controls at 3 dpi (Supplementary Figure S1A,D). Immunostaining of SARS-CoV-2 S protein showed the same expression pattern (Supplementary Figure S1G–I). No expression of SARS-CoV-2 N protein was detected by immunostaining in quadriceps of SARS-CoV-2-, IAV-, or mock-infected hamsters at 3 dpi (Figure 1D–F), 30 dpi, or 60 dpi. TEM did not show any virus-like particles in the quadriceps. H&E staining showed inflammatory cell infiltrates in bronchioles and alveoli in lungs at 3 dpi (Figure 1G) but not in quadriceps at 3 dpi (Figure 1H–J). Therefore, there is no evidence of direct SARS-CoV-2 invasion of skeletal muscle or persistent viral pneumonia in the COVID-19 hamster model.

### 3.2. Respiratory SARS-CoV-2 but Not IAV Infection Induces Skeletal Muscle Atrophy

**Figure 2:**
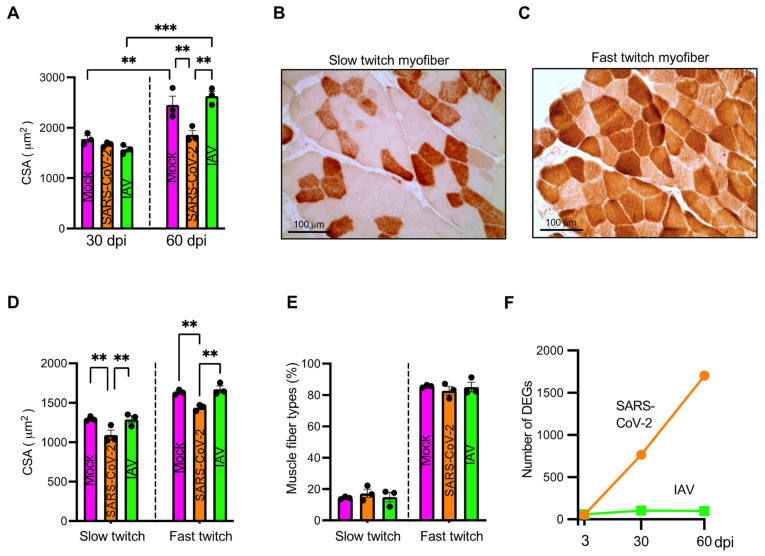
Respiratory infection with SARS-CoV-2 but not influenza A virus (IAV)-induced muscle fiber atrophy. (**A**) Bar graph showing comparison of cross-sectional area (CSA) of myofiber of quadriceps of mock-, SARS-CoV-2-, or IAV-infected hamsters at 30- and 60-days post-infection (dpi). (**B**,**C**) Representative images showing slow twitch myofibers stained intensely by anti-slow skeletal myosin heavy chain antibody (**B**) and fast twitch myofibers stained intensely by anti-fast skeletal myosin heavy chain antibody (**C**). (**D**) Bar graph showing comparison of CSA of slow and fast twitch myofibers of quadriceps from mock-, SARS-CoV-2, or IAV-infected hamsters at 60 dpi. (**E**) Fiber type distribution expressed in relative percent of the total number of myofibers. (**A**,**D**,**E**) Each dot represents mean value of CSA (**A**,**D**) or mean percent of muscle fiber types (**E**) of each hamster. *n* = 3 hamsters/infection group/time point. Data were analyzed by one-way ANOVA with Tukey’s post hoc test and expressed as mean ± SEM. ** *p* < 0.01, *** *p* < 0.001. (**F**) Number of significant differentially expressed genes (DEGs, false discovery rate [FDR] *q* < 0.1) detected by RNAseq of quadriceps muscles derived from SARS-CoV-2 or IAV-infected hamsters at 3, 30, and 60 dpi compared to mock controls. *n* = 3 hamsters/infection group/time point.

To assess histopathological changes in skeletal muscle post-COVID-19, we stained paraffin sections of quadriceps derived from SARS-CoV-2-, IAV-, or mock-infected hamsters at 3, 30, and 60 dpi with H&E. It showed no myofiber necrosis or regeneration, no endomysial inflammation or fibrosis, and no microthrombi. However, myofiber cross-sectional areas (CSA) were significantly smaller in the SARS-CoV-2 group than in the mock and IAV groups at 60 dpi (Figure 2A and Supplementary Figure S2). Since hamsters and their myofiber size grew during the period of 30–60 dpi (14.3–18.5 weeks of age) [21] (Figure 2A), the respiratory SARS-CoV-2 infection might halt the myofiber growth, causing atrophy.

Skeletal muscle consists of two major fiber types with a differential preference for energy metabolism [22]. To address whether oxidative myofibers (slow twitch fibers) and glycolytic myofibers (fast twitch fibers) were differentially affected by the respiratory SARS-CoV-2 infection, we analyzed the CSA of each myofiber subtype at 60 dpi (Figure 2B–E). CSA of both slow and fast twitch myofiber was significantly smaller (*p* < 0.01) in SARS-CoV-2-infected animals than in mock-infected controls (Figure 2D). In contrast, IAV-infected hamsters retained the size of each type of myofiber. The fiber type distribution was not different among the three groups (Figure 2E).

### 3.3. Respiratory SARS-CoV-2 Infection Induces Long-Lasting Downregulation of Skeletal Muscle Genes, Primarily Affecting Oxidative Myofiber Genes

To assess the transcriptional response, we performed RNAseq using quadriceps obtained from SARS-CoV-2-, IAV-, or mock-infected hamsters at 3, 30, and 60 dpi. We performed Wald tests to compare the SARS-CoV-2 or IAV group with the mock group. While the number of significant (FDR *q* < 0.1) differentially expressed genes (DEGs) remained relatively stable in the IAV-infected hamsters over the 60-day period, the number of significant DEGs increased sharply in the SARS-CoV-2-infected hamsters over the same period (Figure 2F), indicating a much greater and longer skeletal muscle transcriptional response to the respiratory SARS-CoV-2 infection than to the IAV infection.

**Figure 3:**
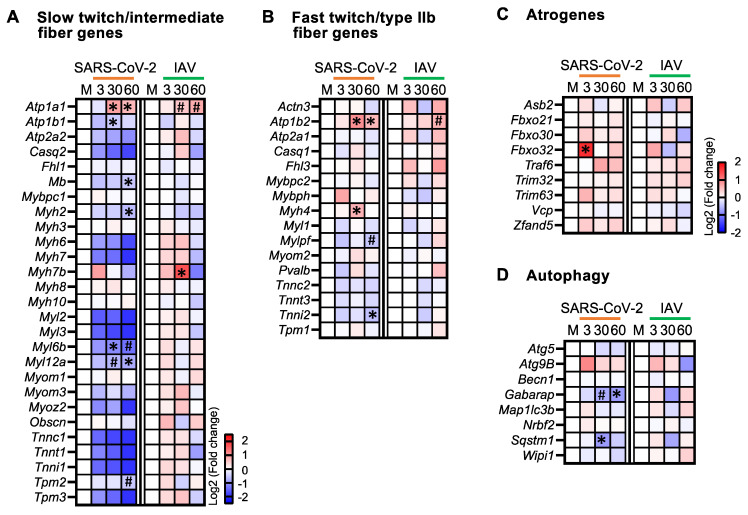
Respiratory SARS-CoV-2 infection induces long-lasting downregulation of oxidative myofiber genes and autophagy genes. (**A**–**D**) Heatmaps showing changes in the expression of slow twitch/intermediate myofiber genes (**A**), fast twitch/type IIb myofiber genes (**B**), atrogenes (**C**), and autophagy genes (**D**) in quadriceps muscles of SARS-CoV-2- and IAV-infected hamsters at different time points compared to mock controls (M). Blue, white, and red indicate log2 (fold change) values of <−2.5, 0, and >2.5, respectively, for (**A**,**B**), and log2 values of <−2, 0, and >2, respectively, for (**C**,**D**). Symbols indicate significant differences compared to mock controls (M) by Wald test (* FDR *q* < 0.05, ^#^ FDR *q* < 0.1). *n* = 3 hamsters/infection group/time point.

Since we observed atrophy in both slow and fast twitch myofibers in SARS-CoV-2-infected hamsters, we analyzed the expression of the genes related to each myofiber subtype [23] (Figure 3A,B). In SARS-CoV-2-infected animals, the majority of the genes that are predominantly expressed by slow/intermediate myofibers were consistently downregulated at all three time points, with several genes achieving statistical significance at 30 and 60 dpi; conversely, no genes were significantly downregulated in IAV-infected animals (Figure 3A). In contrast, the fast/type IIb myofiber genes did not show a consistent pattern of regulation at any time point or infection group, with the exception of a trend of upregulation at 60 dpi in the IAV-infected animals (Figure 3B). These findings indicate that the respiratory SARS-CoV-2 infection primarily affected oxidative fibers at the transcriptional level.

### 3.4. Respiratory SARS-CoV-2 Infection Upregulates Atrogenes and Downregulates Autophagy Genes in Skeletal Muscle

Skeletal muscle fiber size is determined by the balance between protein synthesis and degradation. Because the ubiquitin-proteasome system catalyzes the degradation of most proteins in mammalian cells [24], we assessed the expression of atrogenes involved in this system. In the SARS-CoV-2 group, these genes were upregulated at all three time points, with *Fbxo32* significantly upregulated at 3 dpi (Figure 3C). *Fbxo32* encodes skeletal-muscle-specific E3 ubiquitin ligase F-box protein 32 (FBXO32), which is also known as muscle atrophy F-box (MAFbx) and atrogin-1. It is considered one of the master regulators of muscle atrophy [25]. In contrast, there was not a consistent pattern of regulation in the IAV group, with none of the atrogenes significantly upregulated (Figure 3C). The upregulation of atrogenes in SARS-CoV-2-infected animals might therefore contribute to the muscle atrophy detected.

Another proteolytic system that can contribute to muscle atrophy is autophagy. We assessed the expression of the key genes involved in autophagy initiation (*Becn1*, *Wipi1*, *Atg9b*, *Nrbf2*), autophagosome membrane elongation (*Gabarap*, *Map1lc3β*, *Atg5*), and substrate capture (*Sqstm1*) [26]. While the expressions of *Gabarap* and *Sqstm1* were significantly downregulated in the SARS-CoV-2 group at 30 and 60 dpi, none of these genes showed significant changes in the IAV group (Figure 3D). Therefore, the respiratory infection with SARS-CoV-2, but not IAV, might lead to long-lasting impairment of autophagy in skeletal muscle.

### 3.5. Respiratory SARS-CoV-2 Infection Induces Persistent Downregulation of Genes Involved in Cytoplasmic and Mitochondrial Protein Translation and Mitochondrial Oxidative Phosphorylation (OXPHOS) in Skeletal Muscle

To further characterize the skeletal muscle transcriptional response, we performed GSEA using Wald statistics computed for SARS-CoV-2 and IAV infections at each time point versus mock controls (Supplementary Data S1). There were 415 gene sets with significant coordinate downregulation (normalized enrichment scores [NES] < 0, FDR *q* < 0.1) in the SARS-CoV-2 group relative to the mock group at both 30 and 60 dpi. These included the Reactome “autophagy” pathway, in accordance with the observed downregulation of autophagy genes (Figure 3D), as well as numerous gene sets related to cytosolic and mitochondrial protein translation, mitochondrial function, and oxidative phosphorylation. We thus assessed the genes involved in these processes.

**Figure 4:**
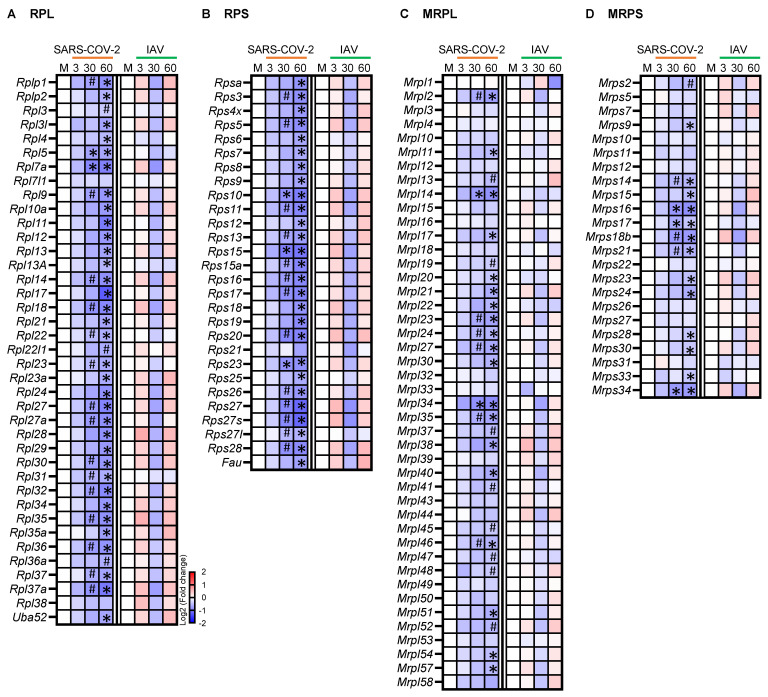
Respiratory infection with SARS-CoV-2 but not influenza A virus (IAV) causes persistent downregulation of many cytosolic and mitochondrial ribosomal protein genes. Heatmaps showing changes in the expression of cytosolic ribosomal protein large subunit (RPL) (**A**), cytosolic ribosomal protein small subunit (RPS) (**B**), mitochondrial ribosomal protein large subunit (MRPL) (**C**), and mitochondrial ribosomal protein small subunit (MRPS) (**D**) genes. Blue, white, and red indicate log2 (fold change) values of <−2, 0, and >2, respectively. Symbols indicate significant differences compared to mock controls (M) by Wald test (* False discovery rate [FDR] *q* < 0.05, ^#^ FDR *q* < 0.1). *n* = 3 hamsters/infection group/time point.

Muscle atrophy can be caused not only by increased protein degradation but also by reduced protein synthesis. Ribosome biogenesis is a fundamental rate-limiting step for protein synthesis, with each ribosome consisting of large and small RNA-protein complexes. In the SARS-CoV-2 group, 17/39 (44%) of the cytosolic ribosomal protein large subunit (RPL) genes and 16/28 (57%) of the small subunit (RPS) genes were significantly downregulated at 30 dpi, and the number of downregulated genes increased to 37/39 (95%) for RPL and 27/28 (96%) for RPS at 60 dpi (Figure 4A,B). In contrast, none of these genes were significantly changed in the IAV group. Thus, the respiratory SARS-CoV-2 infection may induce a unique, long-lasting suppression of cytoplasmic protein translation machinery in skeletal muscle, contributing to muscle atrophy.

We also analyzed the expression of the genes encoding mitochondrial ribosomal proteins (MRPs). Mitochondrial ribosomes translate mRNAs transcribed from mitochondrial DNA, but the MRPs themselves are encoded by nuclear genes. While both large and small subunit MRP genes were consistently downregulated in the SARS-CoV-2 group, with the majority achieving statistical significance at 60 dpi, none of them were significantly changed in the IAV group (Figure 4C,D). Therefore, mitochondrial protein synthesis may also be suppressed in skeletal muscle by a respiratory SARS-CoV-2 infection.

**Figure 5:**
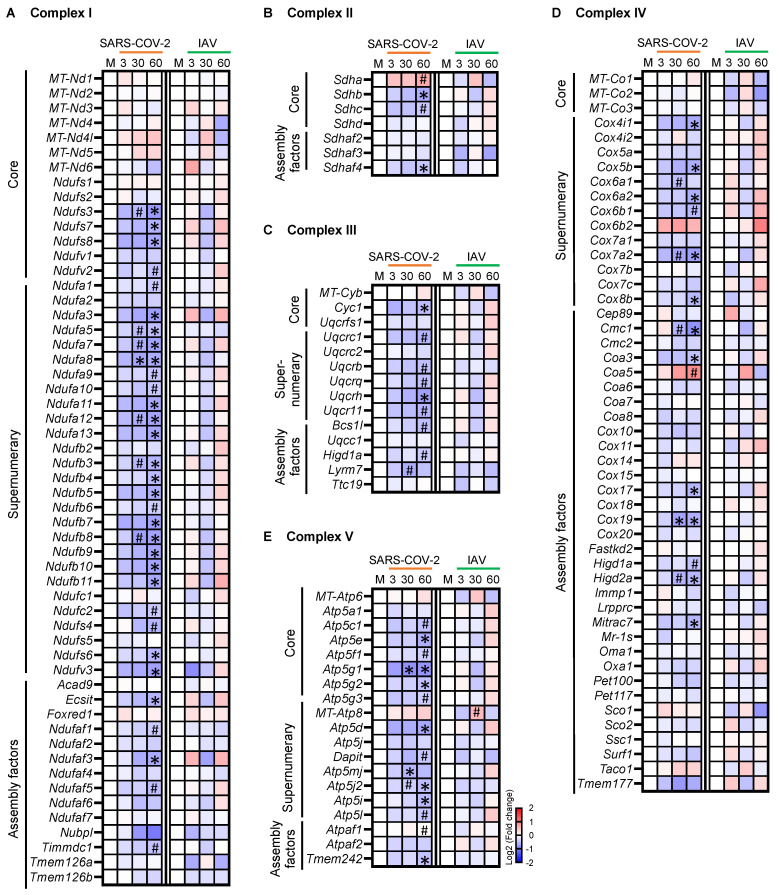
Respiratory infection with SARS-CoV-2 but not influenza A virus (IAV) causes persistent downregulation of nuclear genes encoding core proteins, supernumerary subunit proteins, and assembly factors of all mitochondrial oxidative phosphorylation (OXPHOS) complexes. (**A**–**E**) Heatmaps showing changes in the expression of genes encoding protein components of complex I (**A**), II (**B**), III (**C**), IV (**D**), and V (**E**). Blue, white, and red indicate log2 (fold change) values of <−2, 0, and >2, respectively. Symbols indicate significant differences compared to mock controls (M) by Wald test (* False discovery rate [FDR] *q* < 0.05, ^#^ FDR *q* < 0.1). *n* = 3 hamsters/infection group/time point. MT-: mtDNA genes.

We next analyzed the mRNA expression of the five mitochondrial OXPHOS complexes that are composed of proteins of structural cores, supernumerary subunits, and assembly factors [27], which are encoded by both mtDNA genes and nDNA genes (Figure 5A–E). While the expression of the 13 mtDNA genes encoding essential proteins of complexes I, III, IV, and V did not change significantly in either the SARS-CoV-2 or the IAV group (Figure 5A,C–E), many nDNA genes encoding proteins of all five complexes were significantly downregulated in the SARS-CoV-2 but not the IAV group, with more significant changes seen at 60 dpi than at 30 dpi (Figure 5A–E). Therefore, the respiratory infection with SARS-CoV-2 induced a global and long-lasting downregulation of nuclear genes involved in skeletal muscle OXPHOS.

Mitochondrial function can be affected by altered mitochondrial biogenesis, dynamics (fusion and fission), and mitophagy. To address whether biogenesis was affected at the transcriptional level, we first estimated the relative amount of cellular mtDNA by the ratio of mtDNA/nDNA. The ratio tended to be lower in the SARS-CoV-2 group than in the IAV and mock groups, but the difference was not significant (Supplementary Figure S3A). We then analyzed the expression of the genes involved in mitochondrial biogenesis [28,29,30] (Supplementary Figure S3B). There was not a consistent pattern of differential expression, although at 60 dpi in the SARS-CoV-2 group, the estrogen-related receptor α (ESRRA) gene (*Esrra*) was significantly downregulated and the sirtuin-1 gene (*Sirt1*) was significantly upregulated. ESRRA is a downstream target of peroxisome proliferator-activated receptor-γ coactivator-1 α (PGC-1α), which is the master regulator of mitochondrial biogenesis [31,32]. The transcription of the PGC-1α gene (*Ppagc1a*) itself was not differentially regulated with respect to SARS-CoV-2 infection. None of the main genes involved in the regulation of mitochondrial fusion or fission [29,33] showed significant changes (Supplementary Figure S3C). Among mitophagy pathway genes [29,34], although the expression of PTEN-induced kinase 1 gene (*Pink1*) and parkin RBR E3 ubiquitin protein ligase gene (*Prkn*) did not change significantly, the expression of Bcl2 interacting protein 3 gene (*Bnip3*), *Gabarap1*, and *Sqstm1* were significantly downregulated in the SARS-CoV-2 group at 30 and/or 60 dpi (Supplementary Figure S3D). Taken together, the respiratory SARS-CoV-2 infection inhibited mitophagy and, to a lesser degree, mitochondrial biogenesis, but not mitochondrial fusion or fission at the transcriptional level.

### 3.6. Respiratory SARS-CoV-2 Infection Downregulates Many Enzyme Genes Involved in Fatty Acid β-Oxidation and TCA Cycle

**Figure 6:**
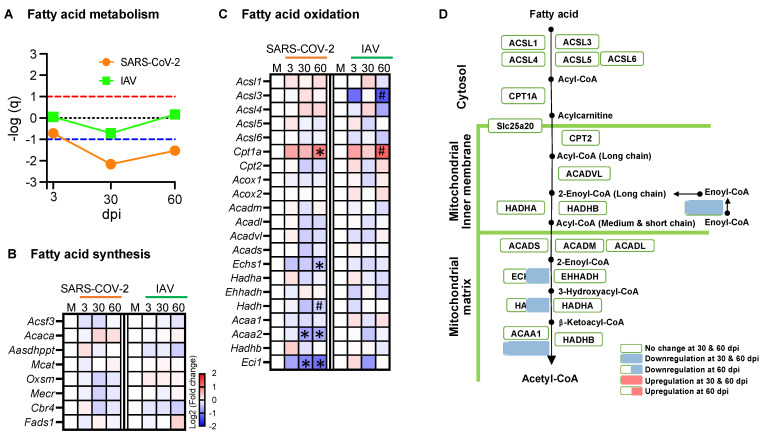
Respiratory SARS-CoV-2 infection downregulates some enzyme genes involved in fatty acid oxidation. (**A**) Summary of Gene Set Enrichment Analysis (GSEA) performed using the Hallmark fatty acid metabolism gene set in SARS-CoV-2 or influenza A virus (IAV)-infected hamsters at 3-, 30-, and 60-days post-infection (dpi) compared to mock controls. Dotted lines indicate false discovery rate (FDR) *q* value = 0.1 for positive (red) and negative (blue) coordinate regulation. *n* = 3 hamsters/infection group/time point. (**B**,**C**) Heatmaps showing changes in the expression of enzyme genes involved in fatty acid synthesis (**B**) and fatty acid oxidation (**C**). (**D**) Schematic overview of expression changes in enzyme genes involved in fatty acid oxidation in the SARS-CoV-2 group. Blue, white, and red indicate log2 (fold change) values of <−2, 0, and >2, respectively. Symbols indicate significant differences compared to mock controls (M) by the Wald test (* FDR *q* < 0.05, ^#^ FDR *q* < 0.1). *n* = 3 hamsters/infection group/time point.

**Figure 7:**
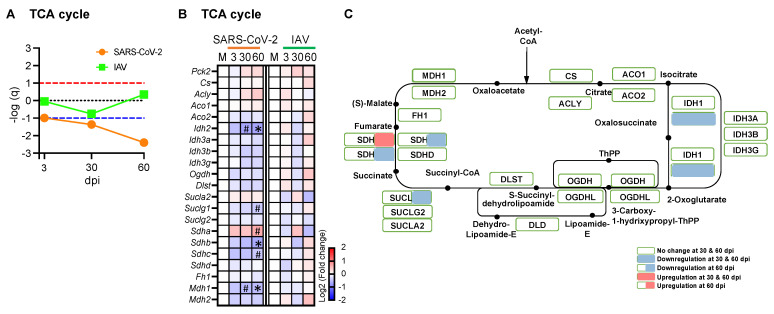
Respiratory SARS-CoV-2 infection downregulates some enzyme genes involved in the TCA cycle. (**A**) Summary of Gene Set Enrichment Analysis (GSEA) performed using the WikiPathways TCA cycle gene set in SARS-CoV-2- or influenza A virus (IAV)-infected hamsters at 3-, 30-, and 60-days post-infection (dpi) compared to mock controls. Dotted lines indicate false discovery rate (FDR) *q* value = 0.1 for positive (red) and negative (blue) coordinate regulation. *n* = 3 hamsters/infection group/time point. (**B**) Heatmap showing changes in the expression of enzyme genes involved in TCA cycle. (**C**) Schematic overview of gene expression changes in TCA cycle enzyme genes in the SARS-CoV-2 group. Blue, white, and red indicate log2 (fold change) values of <−2, 0, and >2, respectively. Symbols indicate significant differences compared to mock controls (M) by Wald test (* FDR *q* < 0.05, ^#^ FDR *q* < 0.1). *n* = 3 hamsters/infection group/time point.

Since the respiratory SARS-CoV-2 infection downregulated OXPHOS genes in skeletal muscle, we next addressed whether the other aspects of the energy metabolism were also affected. To this end, we performed GSEA and analyzed the enzyme genes involved in each catabolic pathway (Figure 6 and Figure 7, and Supplementary Figure S4).

Glycolysis is a cytoplasmic pathway that breaks down glucose into pyruvate. GSEA showed that the genes involved in glycolysis were not coordinately regulated with respect to infection with either SARS-CoV-2 or IAV (Supplementary Figure S4A), although the hexokinase 2 gene (*Hk2*) was significantly upregulated and the lactate dehydrogenase B gene (*Ldhb*) was significantly downregulated in the SARS-CoV-2 group at 30 and 60 dpi (Supplementary Figure S4B,C). In the same manner, the genes participating in amino acid metabolism were not coordinately regulated in response to either infection (Supplementary Figure S4D).

Unlike glycolysis or amino acid metabolism, fatty acid metabolism was coordinately downregulated in the SARS-CoV-2 group at 30 and 60 dpi but not in the IAV group (Figure 6A). While none of the fatty acid synthesis genes showed significant changes (Figure 6B), several genes encoding enzymes involved in the fatty acid β-oxidation showed more significant changes in the SARS-CoV-2 group than in the IAV group at 30 and 60 dpi, among which only the carnitine palmitoyl transferase 1 (CPT1) gene (*Cpt1*) was upregulated at 60 dpi, while the rest were downregulated (Figure 6C,D). β-oxidation is a catabolic process by which fatty acids are broken down to acetyl-CoA. CPT1 is located at the outer mitochondrial membrane to transfer the acyl group from CoA to carnitine, a rate-limiting step in β-oxidation. Within mitochondria, each round of β-oxidation requires the sequential actions of several enzymes, including enoyl-CoA delta isomerase 1 (ECI1), enoyl-CoA hydratase (ECH), hydroxyacyl-CoA dehydrogenase (HADH), and acetyl-Coenzyme A acyltransferase 2 (ACAA2) [35]. The genes encoding these enzymes (*Eci1*, *Echs1*, *Hadh*, and *Acaa2*, respectively) were all significantly downregulated at 60 dpi in the SARS-CoV-2 group but not in the IAV group (Figure 6C,D). Therefore, the respiratory infection with SARS-CoV-2, but not IAV, caused persistent suppression of fatty acid β-oxidation genes.

The TCA cycle is a series of chemical reactions that oxidize acetyl-CoA derived from carbohydrates, lipids, and proteins. GSEA showed significant downregulation of the TCA cycle genes in the SARS-CoV-2 group at 30 and 60 dpi but not in the IAV group (Figure 7A). The isocitrate dehydrogenase gene (*Idh2*), succinate-CoA ligase GDP/ADP-forming subunit alpha gene (*Suclg1*), succinate dehydrogenase genes (*Sdhb* and *Sdhc*), and malate dehydrogenase 1 gene (*Mdh1*) were significantly downregulated by the SARS-CoV-2 infection at 30 and/or 60 dpi (Figure 7B,C). Only *Sdha* was upregulated in the SARS-CoV-2 group at 60 dpi. The findings indicate that the respiratory infection with SARS-CoV-2, but not IAV, induced persistent suppression of the TCA cycle at the transcriptional level.

### 3.7. Respiratory SARS-CoV-2 or IAV Infection Causes Mild Morphological Changes of Intermyofibrillar Mitochondria

**Figure 8:**
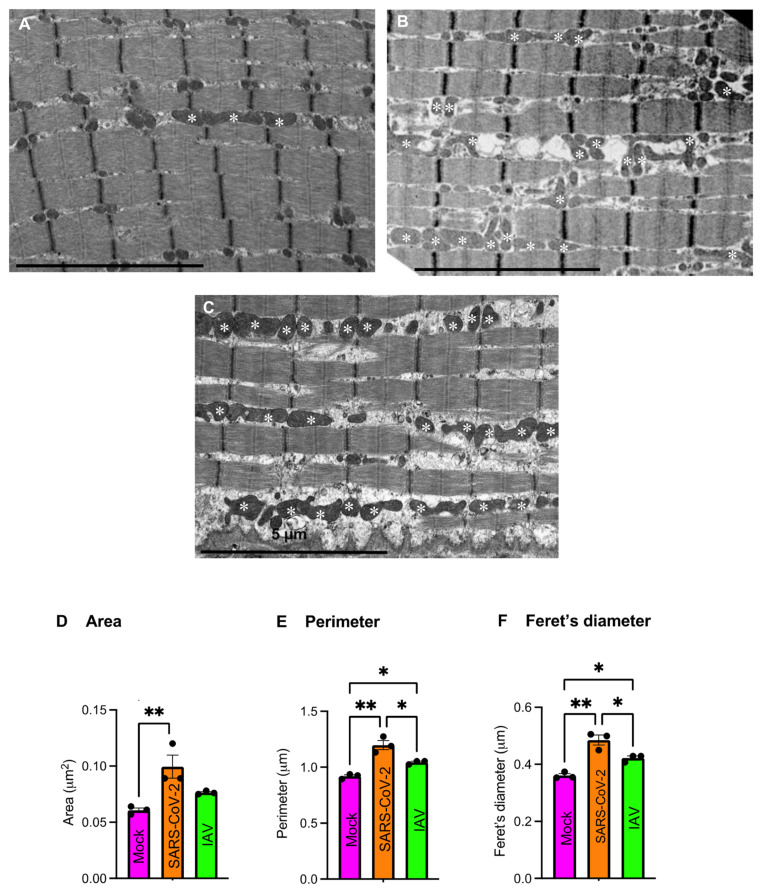
Respiratory SARS-CoV-2 infection causes mitochondrial morphological changes in hamsters and patient. (**A**,**B**) Representative longitudinal electron microscopic images of quadriceps muscle of mock- (**A**) or SARS-CoV-2-infected hamsters (**B**) at day 60 post-infection (dpi) showing an increased number of enlarged and/or elongated mitochondria (white asterisk) in SARS-CoV-2-infected hamsters. (**C**) Longitudinal electron microscopic image of a muscle biopsy from a patient with persistent post-COVID muscle fatigue showing many enlarged and/or elongated mitochondria (white asterisk). (**D**–**F**) Bar graphs showing comparisons of area (**D**), perimeter (**E**), and Feret’s diameter (**F**) of intermyofibrillar (IMF) mitochondria of quadriceps muscles of hamsters at 60 dpi. Each dot represents mean value of area (**D**), perimeter (**E**), and Feret’s diameter (**F**) of each hamster. *n* = 3 hamsters/infection group. Data were analyzed by one-way ANOVA with Tukey’s post hoc test and expressed as mean ± SEM. * *p* < 0.05, ** *p* < 0.01. IAV: Influenza A virus.

Mitochondria are highly dynamic organelles that remodel their shape, size, and distribution to ensure adaptation to cellular bioenergetic requirements and stress. To address whether there were associated changes in mitochondrial morphology, we performed a TEM study. Scattered subsarcolemmal mitochondrial aggregations were similarly observed in both SARS-CoV-2- and mock-infected hamsters, whereas small intermyofibrillar mitochondrial aggregations were more frequently seen in the SARS-CoV-2 group than in the mock group. In the SARS-CoV-2 group, mitochondrial cristae appeared normal with no inclusions, but focal loss of myofilaments was observed. Enlarged and elongated mitochondria were more frequently seen in SARS-CoV-2 hamsters than in mock-infected controls (Figure 8A,B). We assessed the size and shape of subsarcolemmal (SS) and intermyofibrillar (IMF) mitochondria in quadriceps muscles. IMF mitochondria, but not SS mitochondria (Supplementary Figure S5A–D, Supplementary Table S2), were morphologically different among 3 groups. The IMF mitochondria in the SARS-CoV-2 group were larger in area, perimeter, and Feret’s diameter than those in the IAV and mock groups (Figure 8D–F, Supplementary Table S3), indicating that the IMF mitochondria in the SARS-CoV-2 group are more enlarged and elongated, a sign of mitochondria stress [36,37,38]. Similar changes in mitochondria were seen in a muscle biopsy from a young adult patient who experienced persistent myalgia and fatigue following COVID-19 with no other causes identified (Figure 8C).

### 3.8. Respiratory SARS-CoV-2 Infection Induces Type I and Type II Interferon (IFN) Responses and Tumor Necrosis Factor-Alpha (TNF-α) Response in Skeletal Muscle

**Figure 9:**
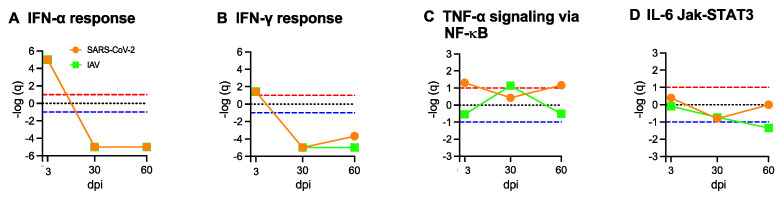
Type I and Type II interferon and TNF-α/NFκB cytokine responses are induced in muscle by respiratory SARS-CoV-2 infection. Summary of Gene Set Enrichment Analysis (GSEA) performed using Hallmark cytokine/inflammation gene sets in SARS-CoV-2- or Influenza A virus (IAV)-infected hamsters at 3-, 30-, and 60-days post infection (dpi) compared to mock controls. Dotted lines indicate false discovery rate (FDR) *q* value = 0.1 for positive (red) and negative (blue) coordinate regulation. *n* = 3 hamsters/infection group/time point. Results are shown for IFN-α response (**A**), IFN-γ response (**B**), TNF-α/NF-κB signaling response (**C**), and IL-6/JAK-STAT3 signaling response (**D**).

Acute respiratory SARS-CoV-2 infection generates a robust systemic cytokine response in addition to type I and type II interferon responses, and the plasma levels of IFN-α, IFN-γ, IL-1β, IL-6, and TNF-α are significantly increased in human patients with acute COVID-19 [39,40,41,42,43]. Since there is no evidence of direct SARS-CoV-2 viral invasion, we used GSEA to assess whether the transcriptional changes observed in skeletal muscle were triggered by the systemic responses. Both SARS-CoV-2 and IAV respiratory infections induced acute and transient type I and type II interferon responses in skeletal muscle, with strong coordinated upregulation of IFN-α and IFN-γ response genes at 3 dpi but not at 30 or 60 dpi (Figure 9A,B). Likewise, IFN-α and IFN-γ responses were strongly induced at 3 dpi but not at 30 dpi in the lungs (Supplementary Figure S6A,B). TNF-α signaling via NFκB was coordinately upregulated in muscles at 3 and 60 dpi in the SARS-CoV-2 group, but only at 30 dpi in the IAV group (Figure 9C), although the expression of the TNF-α gene (*Tnf*) itself was extremely low in muscles. In the lungs, TNF-α signaling via NFκB was strongly enriched at 3 dpi and then very low at 30 dpi in both SARS-CoV-2 and IAV groups (Supplementary Figure S6C). While IL-6 signaling was strongly induced in the lungs following acute respiratory SARS-CoV-2 infection (Supplementary Figure S6D), it was not coordinately upregulated in skeletal muscle (Figure 9D). These findings suggest an interesting hypothesis that the persistent transcriptional changes in the skeletal muscle of SARS-CoV-2-infected animals might be related to the transient systemic interferon and TNF-α responses during the acute viral infection.

### 3.9. Co-Treatment of C2C12 Myotubes with IFN-α, IFN-γ, and TNF-α Markedly Impairs Mitochondrial Respiration and Shifts Energy Metabolism from Oxidative Respiration to Glycolysis

**Figure 10:**
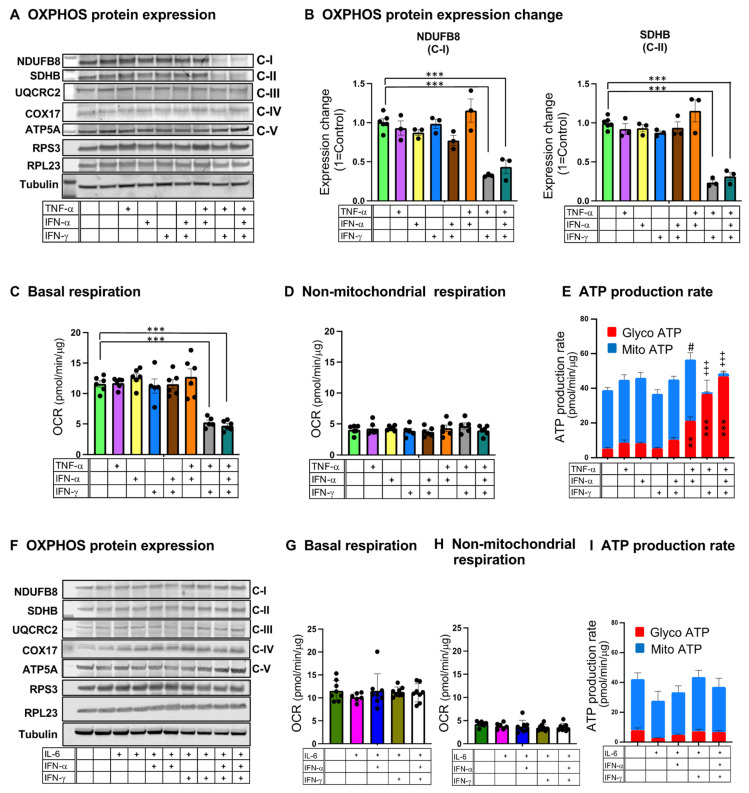
Treatment with a combination of Type II interferon and TNF-α leads to mitochondrial dysfunction in C2C12 myotubes. (**A**–**E**) Effects of treatments of C2C12 myotubes with IFN-α, IFN-γ, and TNF-α individually or in combinations. (**A**) Immunoblot analysis of OXPHOS protein expression. (**B**) Quantification of NADH dehydrogenase 1 beta subcomplex subunit 8 (NDUFB8, Complex I; C-I) and succinate dehydrogenase complex iron sulfur subunit B (SDHB, Complex II; C-II) protein expression normalized to tubulin and relative to untreated control. Data were analyzed by one-way ANOVA with Tukey’s post hoc test and expressed as mean ± SEM of three independent experiments. (**C**) Oxygen consumption rate under basal conditions (basal respiration). (**D**) Non-mitochondrial respiration. (**E**) Glycolytic (Glyco) ATP production rate (red) and mitochondrial (Mito) ATP production rate (blue). (**F**–**I**) Effects of treatments of C2C12 myotubes with IFN-α, IFN-γ, and IL-6 individually or in combinations. (**F**) Immunoblot analysis of OXPHOS protein expression. (**G**) basal respiration. (**H**) non-mitochondrial respiration. (**I**) Glycolytic (Glyco) ATP production rate (red) and mitochondrial (Mito) ATP production rate (blue). For (**C**–**E**,**G**–**I**), data were analyzed by one-way ANOVA with Tukey’s post hoc test and expressed as mean ± SEM of 3 repetitive experiments with 5 or 6 technical replicates. ** *p* < 0.01, *** *p* < 0.001, ^#^ *p* < 0.05, ^+++^ *p* < 0.001. + indicates treatment with IFN-α, IFN-γ, TNF-α, or IL-6.

To address whether the combination of interferons and TNF-α could potentially trigger skeletal muscle abnormalities seen in the COVID-19 hamster model, we treated differentiated C2C12 myogenic cells (myotubes) with IFN-α, IFN-γ, and TNF-α individually or in various combinations. Immunoblot analysis showed a significant reduction in NADH dehydrogenase 1 beta subcomplex subunit 8 (NDUFB8, complex I) and succinate dehydrogenase complex iron sulfur subunit B (SDHB, complex II) protein expression after 48 h of treatment with IFN-α/IFN-γ/TNF-α or IFN-γ/TNF-α but not with interferon or TNF-α alone (Figure 10A,B). The expression of selected proteins of complexes III, IV, and V, as well as ribosome small and large units, did not show changes with treatments (Figure 10A and Supplementary Figure S7A,B). To assess mitochondrial oxidative function, we measured the oxygen consumption rate (OCR) using a Seahorse cell metabolic analyzer. Twenty-four hours of treatment with IFN-α/IFN-γ/TNF-α or IFN-γ/TNF-α, but not the others, dramatically reduced basal respiration rate (Figure 10C), but non-mitochondrial respiration did not change by any treatment (Figure 10D). The treatments did not change the total protein amount compared to untreated controls (Supplementary Figure S7C), indicating that the reduction in basal respiration was not caused by cell death. We also measured ATP production rates by mitochondrial oxidation and glycolysis. C2C12 myotubes displayed high oxidative metabolism at baseline; however, IFN-α/IFN-γ/TNF-α and IFN-γ/TNF-α treatments induced a dramatic shift from oxidative respiration towards glycolysis (Figure 10E). The combinations of IL-6 with interferons, including IFN-α/IFN-γ/IL-6 and IFN-γ/IL-6, did not affect OXPHOS protein expression, basal respiration, or oxidative and glycolytic ATP production rates (Figure 10F–I). Therefore, the combination of type II interferon with TNF-α but not IL-6 impaired mitochondrial oxidative functions in skeletal muscle cells in vitro.

## 4. Discussion

Muscle fatigue represents the most common symptom that persists after COVID-19. By characterizing the longitudinal skeletal muscle histopathological and transcriptional changes after acute respiratory SARS-CoV-2 infection in the COVID-19 hamster model, our present study has generated several important findings that shed light on the potential mechanisms underlying muscle symptoms associated with COVID-19 and long COVID.

First, our study supports the notion that SARS-CoV-2 is unlikely to directly invade skeletal muscle after an acute respiratory infection. So far, there has been no convincing evidence that SARS-CoV-2 directly invades skeletal muscle in humans [12,14,44,45]. In the COVID-19 hamster model, our present study shows no evidence of direct SARS-CoV-2 infection of skeletal muscle, as SARS-CoV-2 RNA and protein expression, virus-like particles, and inflammatory cell infiltrates are all absent in skeletal muscle.

Second, despite the absence of direct viral invasion, skeletal muscle in the COVID-19 hamster model undergoes myofiber atrophy and long-lasting transcriptomic changes, which are not observed with acute respiratory IAV infection. Myofiber atrophy has also been reported in muscle biopsies of patients with long COVID [11,12,13]. Our study further shows that both oxidative and glycolytic myofibers undergo atrophy, which argues against immobilization being the sole cause of the atrophy, as disuse has a primary impact on glycolytic fibers [46]. In parallel with this muscle atrophy, atrogenes are upregulated while many cytoplasmic ribosomal protein genes are downregulated, suggesting that the myofiber atrophy is likely a result of both accelerated protein degradation and impaired protein synthesis.

Another prominent transcriptional response detected in skeletal muscle after respiratory SARS-CoV-2 infection is the long-lasting suppression of genes related to mitochondrial energy metabolism, especially those involved in mitochondrial OXPHOS, fatty acid β-oxidation, and the TCA cycle. Consistent with our findings, reduced expression of OXPHOS proteins, impaired mitochondrial respiration, and altered muscle metabolism with a lower reliance on oxidative metabolism have been observed in patients with exercise intolerance associated with long COVID [13,14]. Our study further shows that the respiratory SARS-CoV-2 infection affects mitochondrial oxidative metabolism at the nuclear gene level, as the 13 protein-encoding mtDNA genes are not affected.

Third, the systemic cytokine response to acute respiratory SARS-CoV-2 infection is likely an important trigger of the persistent histopathological and transcriptional changes observed in skeletal muscle. While respiratory SARS-CoV-2 and IAV infections generate comparable acute and transient type I and II interferon responses in skeletal muscle, the inflammatory cytokine response is different, with the TNF-α/NF-κB signaling pathway being differentially upregulated in the former. SARS-CoV-2 can infect cells and bind to critical host mitochondrial proteins to inhibit mitochondrial function [47,48]. The impaired mitochondrial functions can persist in a variety of non-muscle tissues, even after the virus is cleared [49]. Our present study further shows that the respiratory SARS-CoV-2 infection can persistently suppress mitochondrial oxidative metabolism genes in skeletal muscle without direct infection, which suggests a role of the acute systemic responses in the pathogenesis of muscle abnormalities. There is no evidence of persistent viral pneumonia or a chronic systemic response in our model.

The host interferon response is critical for controlling viral infection, but it can also enhance the inflammatory cytokine response. Exuberant systemic inflammatory cytokine response is a prominent feature of acute respiratory SARS-CoV-2 infection [40]. The plasma levels of type I and type II interferons as well as several inflammatory cytokines, including IFN-α, IFN-γ, IL-6, TNF-α, and IL-1β, are significantly increased in human patients during acute infection [39,40,41,42,43]. While IL-6 signaling is strongly induced in the lungs following acute respiratory SARS-CoV-2 infection [15,40], the genes in this pathway are not coordinately regulated in skeletal muscle, as shown by our transcriptome study. Genes involved in TNF-α/NF-κB signaling, however, are coordinately upregulated in muscle at the acute phase. Given the finding that TNF-α ligand expression is extremely low in skeletal muscle, the circulating TNF-α may act on skeletal muscle to cause muscle abnormalities. The TNF-α/NF-κB signaling pathway is known to induce skeletal muscle atrophy [50,51,52] by inhibiting muscle protein synthesis and increasing protein breakdown [53]. Activation of TNF-α/NF-κB signaling can also lead to upregulation of *Fbxo32* [54], which is significantly upregulated in skeletal muscle at day 3 post-respiratory SARS-CoV-2 infection. Therefore, the enrichment of this signaling pathway likely contributes to muscle atrophy.

Myalgia and fatigue are common side effects of IFN-α treatment in patients with hepatitis C, which can lead to chronic fatigue. Comparing with healthy controls, one study reported that the patients who developed chronic fatigue after IFN-α treatment showed high serum levels of IL-6 and TNF-α during but not after the treatment [55]. The findings lead us to speculate that although the type I and type II interferon responses are transient in skeletal muscle after the respiratory SARS-CoV-2 infection, the combination of the systemic interferon and TNF-α responses during acute infection might exert a synergistic impact on skeletal muscle and set the stage for chronic muscle fatigue. Importantly, the simultaneous upregulation of IFN-α, IFN-γ, and TNF-α was observed only in SARS-CoV-2-infected hamsters but not in IAV-infected hamsters. This difference might contribute, in part, to the different impact on mitochondrial oxidative function and the persistency of the abnormality. In support of this notion, our in vitro study showed that the treatment of C2C12 myotubes with combined IFN-γ and TNF-α but not IFN-γ or TNF-α alone markedly impaired mitochondrial oxidative function. Although our in vitro study did not demonstrate a significant impact of IFN-α on mitochondrial oxidative function, IFN-α might still play a role in vivo, as this response was also significantly upregulated by the acute SARS-CoV-2 infection. Future studies are needed to further elucidate the mechanisms. Our findings suggest that targeting TNF-α during acute SARS-CoV-2 infection may be beneficial to the prevention or mitigation of persistent muscle fatigue. Drugs, which can boost mitochondrial functions, enhance protein synthesis, and inhibit protein degradation, may also be useful for treating muscle fatigue associated with long COVID.

Our study has several limitations. Our cohort is relatively small and may not have sufficient statistical power to detect all the abnormalities in skeletal muscle. Since no fresh specimens could be withdrawn from the BSL-3 laboratory for serum or tissue protein assays such as ELISA and Western blot, we were unable to assess serum cytokines or muscle proteins to correlate with the transcriptional changes in the hamsters. Many proteins that regulate skeletal muscle atrophy and energy metabolism are activated at the translational and post-translational levels, alterations of which cannot be detected by our transcriptional study. Nevertheless, our study is informative and may help guide future studies and therapy development. The hamster model appears valuable for future studies of muscle abnormalities associated with COVID-19 and long COVID, given the significant histopathological and transcriptional changes detected.

## Data Availability

Raw and processed RNAseq data have been deposited in the Gene Expression Omnibus (GEO) and are accessible through GEO Series accession number GSE231910. This paper does not report the original code. Any additional information required to reanalyze the data reported in this paper is available upon request to the corresponding author (shomma@bu.edu).

## Supplementary Materials

The following supporting information can be downloaded at: https://www.mdpi.com/article/10.3390/biomedicines12071443/s1. Figure S1: Robust expression of SARS-CoV-2 protein in the lung of a SARS-CoV-2 respiratory infected hamster at 3-days post infection (dpi), but no positive expression at 30 dpi; Figure S2: Respiratory infection with SARS-CoV-2 but not IAV-induced skeletal muscle fiber atrophy; Figure S3: Respiratory SARS-CoV-2 infection does not impact the relative amount of mitochondrial DNA, mitochondrial biogenesis genes, or fusion and fission genes, but downregulates some mitophagy genes; Figure S4: Respiratory SARS-CoV-2 infection has mild effects on the expression of genes involved in glycolysis and amino acid metabolism; Figure S5: Limited effects of respiratory SARS-CoV-2 or IAV infection on the morphology of subsarcolemmal mitochondria; Figure S6: Type I and Type II interferon responses, TNF-α/NF-κB and IL6/JAk/STAT3 are induced by SARS-CoV-2 infection at acute phase but not post-recovery phase in lungs; Figure S7: Limited effects of inflammatory cytokine treatments on expressions of some OXPHOS complex and ribosomal proteins in C2C12 myotubes; Table S1: qRT-PCR primer sequences; Table S2: Comparisons of morphometric and shape descriptors of subsarcolemmal mitochondria; Table S3: Comparisons of morphometric and shape descriptors of intermyofibrillar mitochondria; Data S1: Summary of Gene Set Enrichment Analysis (GSEA) results.
