# Supplementary material for: Respiratory SARS-CoV-2 Infection Causes Skeletal Muscle Atrophy and Long-Lasting Energy Metabolism Suppression
Source: Biomedicines. 2024 Jun 28;12(7):1443. doi: 10.3390/biomedicines12071443 (PMC11275164; doi:10.3390/biomedicines12071443)
Supplement: Supplementary file 1 [file biomedicines-12-01443-s001.zip › biomedicines-3040396-supplementary.pdf]

Supplementary materials for

Respiratory SARS-CoV-2 Infection Induces Skeletal Muscle Atrophy and Long-Lasting Energy Metabolism Suppression

Sachiko T. Homma[1*], Xingyu Wang[2], Justin J. Frere[3], Adam C. Gower[4], Jingsong Zhou[5], Jean K. Lim[3], Benjamin R. tenOever[6], Lan Zhou[2*]

[1] Department of Neurology, Boston University Chobanian & Avedisian School of Medicine, Boston, MA, 02118, USA.

[2] Department of Neurology, Hospital for Special Surgery, New York, NY, 10021, USA.

[3] Department of Microbiology, Icahn School of Medicine at Mount Sinai, New York, NY, 10029, USA.

[4] Clinical and Translational Science Institute, Boston University Chobanian & Avedisian School of Medicine, Boston, MA, 02118, USA.

[5] College of Nursing and Health Innovation, University of Texas at Arlington, Arlington, TX, 76010, USA.

[6] Department of Microbiology, New York University Grossman School of Medicine, New York, NY, 10029, USA.

The PDF file includes:

Supplementary Figure S1-7

Supplementary Table S1-3

Other Supplemental Material for this manuscript includes the following:

Supplementary Data S1

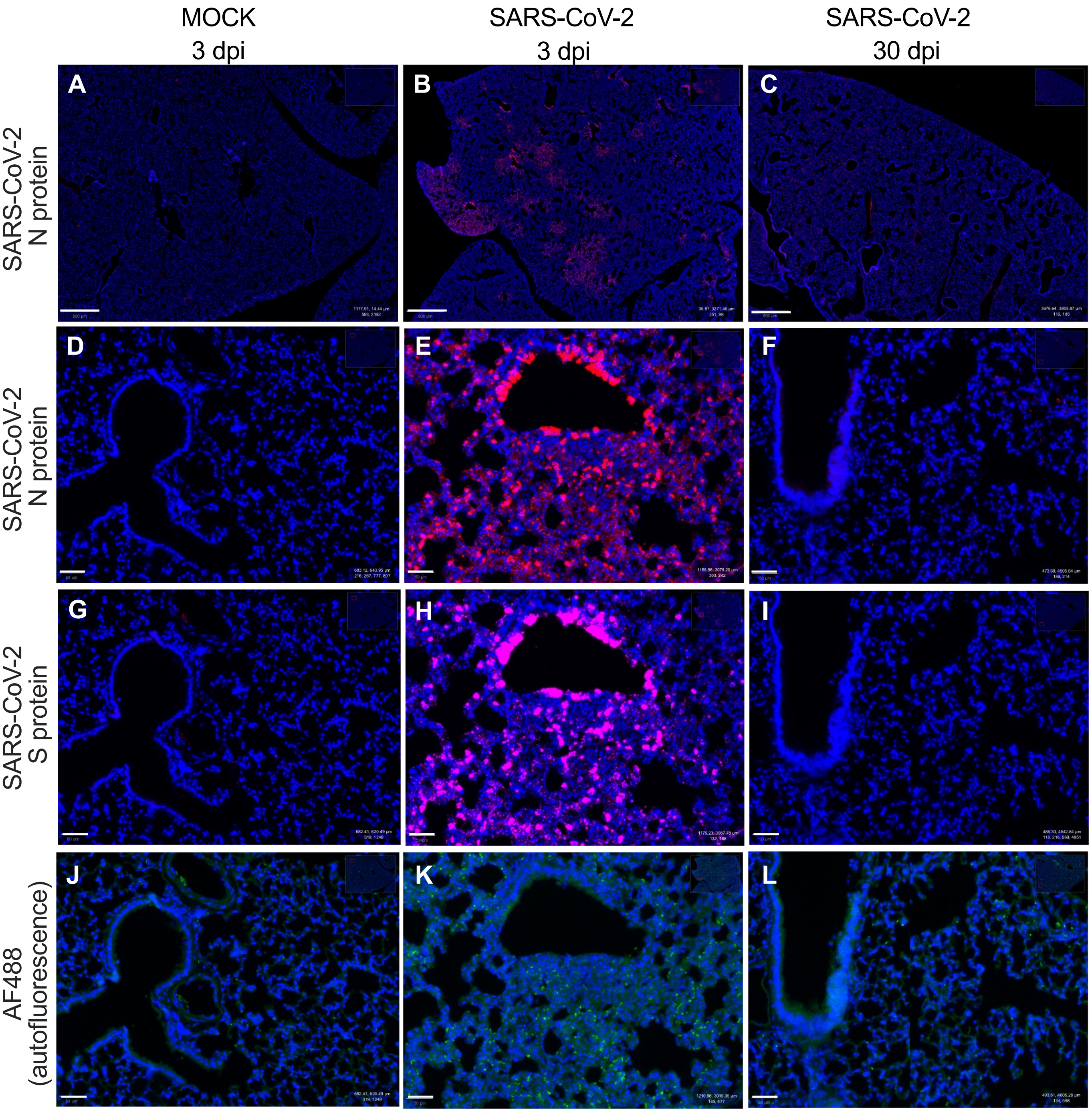

Supplementary Figure S1. Robust expression of SARS-CoV-2 protein in lung of SARS-CoV-2 respiratory infected hamster at 3-days post infection (dpi) but no positive expression at 30 dpi. (A-F) SARS-CoV-2 N protein staining on lung tissues from mock-infected hamster at 3 dpi (A,D), SARS-CoV-2 respiratory infected hamsters at 3 dpi (B,E), and 30 dpi (C,F). SARS-CoV-2 N protein; red fluorescence (Alexa Fluor 568), DAPI; blue fluorescence. Bar = 800 μm (A-C) and 50 μm (D-F). (G-I) SARS-CoV-2 S protein staining on lung tissues from mock-infected hamster at 3 dpi (G), SARS-CoV-2 respiratory infected hamsters at 3 dpi (H), and 30 dpi (I). SARS-CoV-2 S protein; pink fluorescence (Alexa Fluor 647), DAPI; blue fluorescence. Bar = 50 μm (G-I). (J-L) Autofluorescence captured by green fluorescence channel to monitor false positive cells for SARS-CoV-2 protein. Lung tissues from mock infected hamster at 3 dpi (J), SARS-CoV-2 respiratory infected hamsters at 3 dpi (K), and 30 dpi (L). Autofluorescence; green fluorescence, DAPI; blue fluorescence. Bar = 50 μm (J-L). Representative images from n = 3 hamsters/infection group/time point.

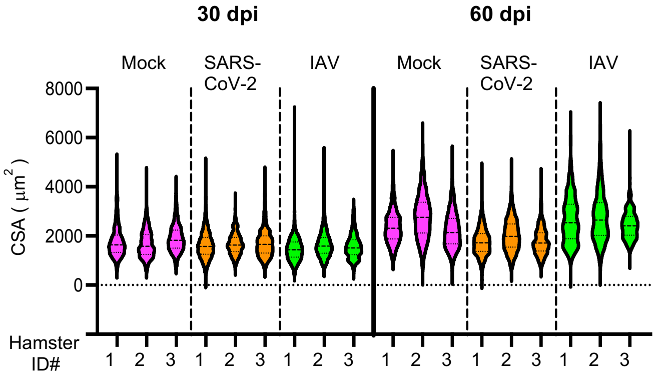

Supplementary Figure S2. Respiratory infection with SARS-CoV-2 but not IAV induces skeletal muscle fiber atrophy. Violin plots showing cross sectional area (CSA) of myofibers of quadriceps muscle from mock-, SARS-CoV-2-, or IAV-infected hamster at 30 and 60 day of post infection (dpi).

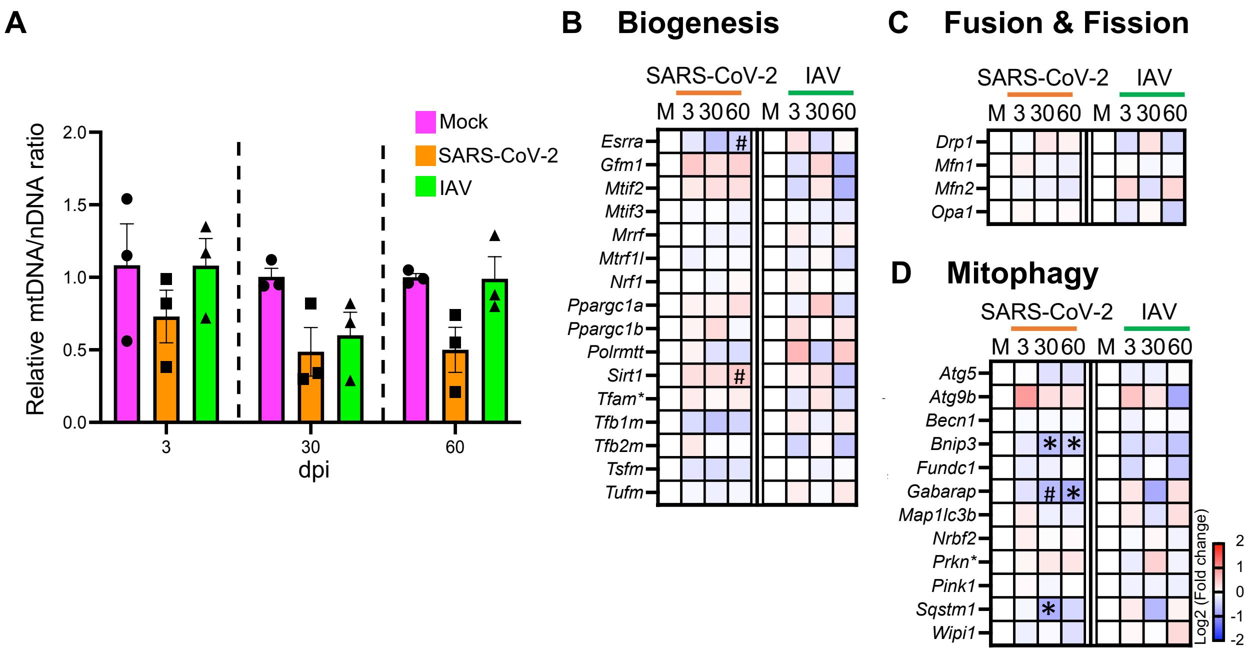

Supplementary Figure S3. Respiratory SARS-CoV-2 infection does not impact relative amount of mitochondrial DNA, mitochondrial biogenesis genes, or fusion and fission genes, but downregulates some mitophagy genes. (A) Bar graph showing amount of mitochondrial DNA gauged by mtDNA/nDNA ratio and expressed as relative value to mock controls at 3-, 30-, and 60-days post infection (dpi). Data shows mean ± SEM, *n* = 3 hamsters/infection group/time point. (B-D) Heatmaps showing changes in the expression of genes involved in mitochondrial biogenesis (B), mitochondrial fusion & fission (C), and mitophagy (D). Blue, white, and red indicate log2 (fold change) values of < -2, 0, and > 2, respectively. Symbols indicate significant differences compared to mock controls (M) by Wald test (*False discovery rate [FDR] $q < 0.05$, $^{\#}$FDR $q < 0.1$). *n* = 3 hamsters/infection group/time point. Fold changes were computed as follows for genes *Tfam* and *Prkn*, which are not present in the genome build used in this study (MesAur1.0): read pairs were aligned to RefSeq records XM_040732203.1 (*Tfam*) and XM_040758174.1 (*Prkn*) using Bowtie (version 2.3.4.1) with local alignment, trimming off the last base of each read, and the number of aligned read pairs was normalized to the total number of read pairs to generate reads per million (RPM) values, which were then used to compute fold changes. No significance tests were performed for these two genes.

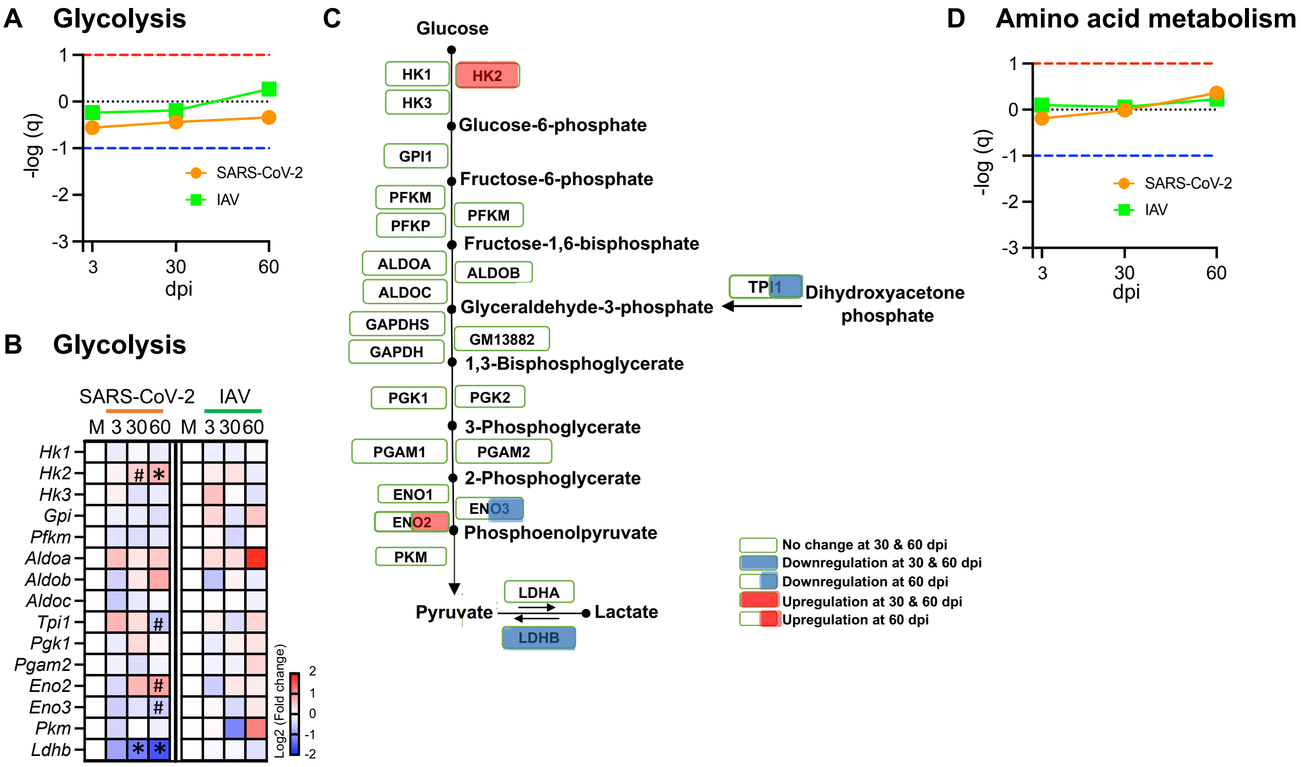

Supplementary Figure S4. Respiratory SARS-CoV-2 infection has mild effects on expression of genes involved in glycolysis and amino acid metabolism. (A) Summary of Gene Set Enrichment Analysis (GSEA) performed using the Hallmark glycolysis gene set in SARS-CoV-2, or IAV-infected hamsters at 3-, 30-, and 60-days infection (dpi) compared to mock controls. Dotted lines indicate false discovery rate (FDR) q value = 0.1 for positive (red) and negative (blue) coordinate regulation. n = 3 hamsters/infection group/time point. (B) Heatmap showing changes in the expression of enzyme genes involved in glycolysis. Blue, white, and red indicate log2 (fold change) values of < -2, 0, and > 2, respectively. Symbols indicate significant differences compared to mock controls (M) by Wald test (*FDR $q < 0.05$, $^{\#}$FDR $q < 0.1$). n = 3 hamsters/infection group/time point. (C) Schematic overview of expression changes of glycolysis enzyme genes in the SARS-CoV-2 group. (D) Summary of GSEA performed using the Hallmark amino acid metabolism gene set in SARS-CoV-2, or IAV-infected hamsters at 3, 30, and 60 dpi compared to mock controls. n = 3 hamsters/infection group/time point.

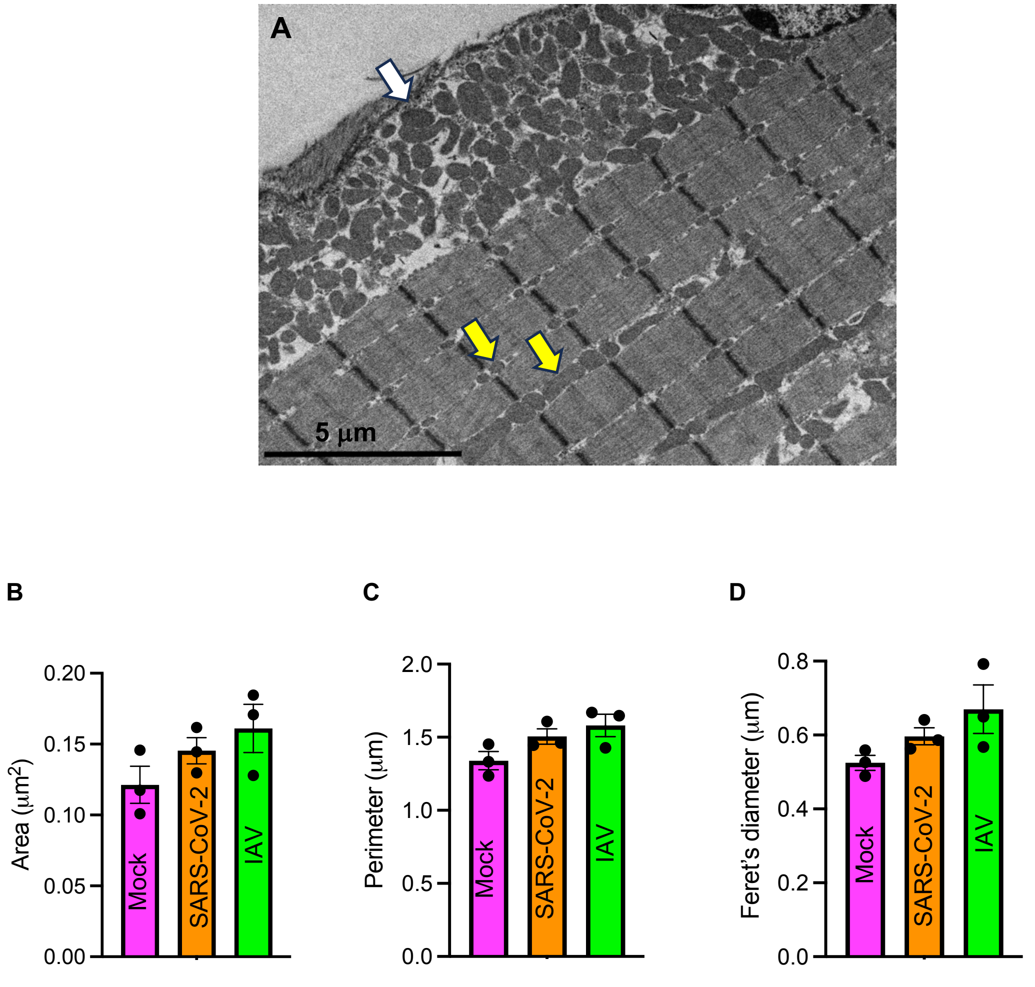

Supplementary Figure S5. Limited effect of respiratory SARS-CoV-2 or IAV infection on morphology of subsarcolemmal mitochondria. (A) Representative longitudinal electron microscopic images of quadriceps muscle of SARS-CoV-2-infected hamsters at 60-days post infection (dpi) showing subsarcolemmal (SS, example; white arrow) and intermyofibrillar (IMF, example; yellow arrows) mitochondria aggregation. (B-D) Bar graphs showing comparisons of morphological parameters and shape descriptors of SS mitochondria at 60 dpi. Area (B), perimeter (C), and Feret's diameter (D). Data are expressed as mean ± SE and analyzed by one-way ANOVA with Tukey's post hoc test. *n* = 3 hamsters/infection group/time point.

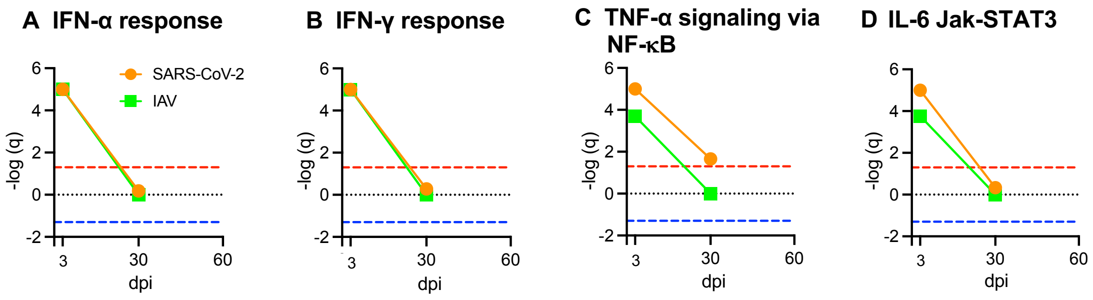

Supplementary Figure S6. Type I and Type II interferon responses, TNF-α/NFκB, and IL6/JAK/STAT3 are induced by SARS-CoV-2 infection at acute phase but not post-recovery phase in lungs. Summary of Gene Set Enrichment Analysis (GSEA) performed using cytokine/inflammation gene sets for lung tissues of SARS-CoV-2- or IAV-infected hamsters at 3- and 30-days post infection (dpi) compared to mock controls. Dotted lines indicate False discovery rate (FDR) *q* value = 0.1 for positive (red) and negative (blue) coordinate regulation. *n* = 3 hamsters/infection group/time point.

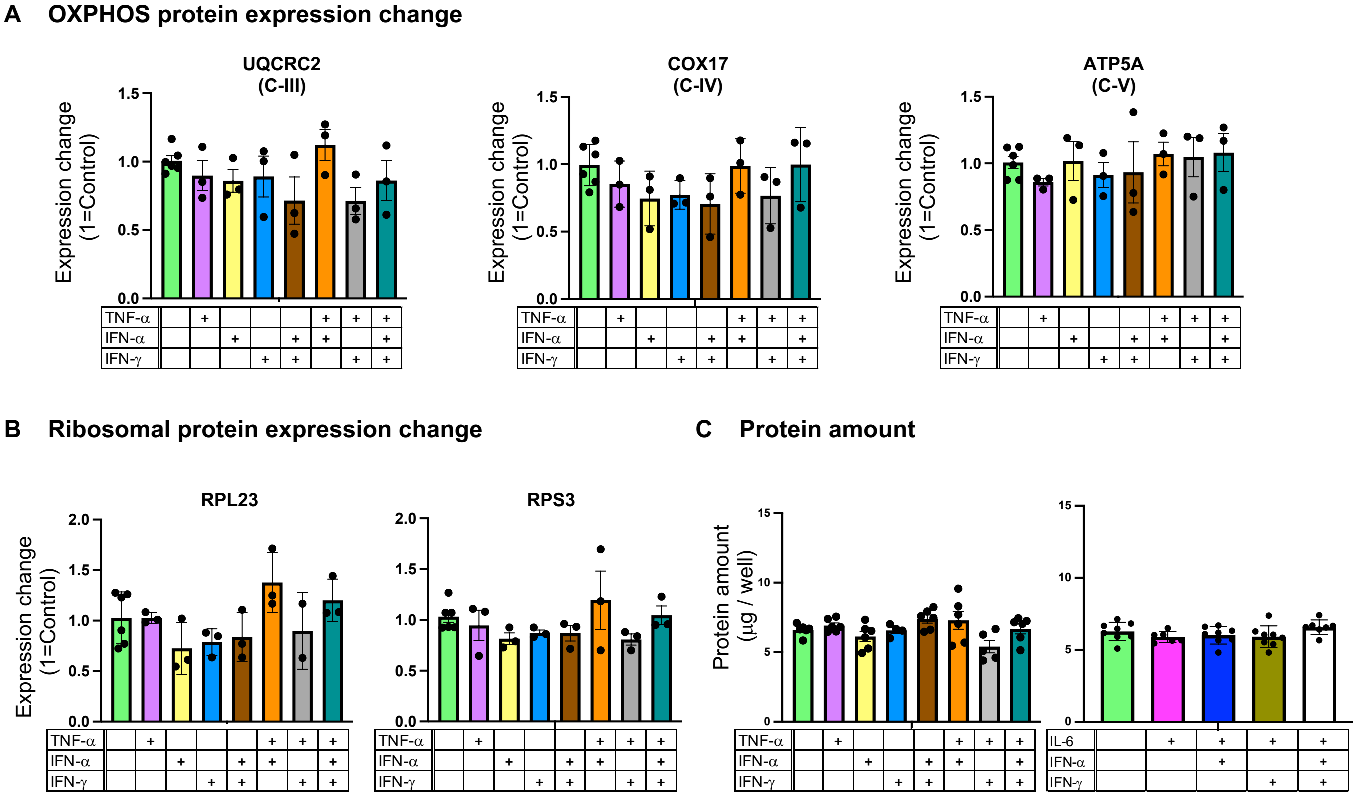

Supplementary Figure S7. Limited effects of inflammatory cytokine treatments on expressions of some OXPHOS complex, and ribosomal proteins in C2C12 myotubes. (A-B) Bar graphs showing protein expression change of UQCRC2 (Complex III; C-III), COX17 (Complex IV; C-IV), and ATP5A (Complex V; C-V) (A), and ribosomal protein large subunit 23 (RPL23) and ribosomal protein small subunit 3 (RPS3) (B). Data are expressed mean ± SEM of 3 independent experiments and analyzed by one-way ANOVA with Tukey's post hoc test. (C) Protein amount. Representative data of 3 repetitive experiments with 5 or 6 technical replicates are shown. Data are expressed as mean ± SEM and analyzed by one-way ANOVA with Tukey's post hoc test.
.

Supplementary Table S1. qRT-PCR primer sequences

Primer name	Direction	Sequence
SARS-CoV-2 Nsp14	Forward	TGGGGYTTTACRGGTAACCT
SARS-CoV-2 Nsp14	Reverse	AACRCGCTTAACAAAGCACTC
SARS-CoV-2 sgN (TRS-L)	N/A	CTCTTGTAGATCTGTTCTCTAAACGAAC
SARS-CoV-2 sgRNA (TRS-N)	N/A	GGTCCACCAAACGTAATGCG
M.auratus ND1	Forward	GCCACCTCAACATCCCTATTT
M.auratus ND1	Reverse	AGTCATGTGTGCTCTTGTGTA
M.auratus ACTB	Forward	CACCATTGGCAACGAGCGGTTC
M.auratus ACTB	Reverse	AGGTCTTTGCGGATGTCGACGT

N/A: not applicable

Supplementary Table S2. Comparisons of morphometric and shape descriptors of subsarcolemmal mitochondria

	Mock			SARS-CoV-2			IAV		
Animal ID	#1	#2	#3	#1	#2	#3	#1	#2	#3
Area Mean (μm)	0.146	0.117	0.101	0.162	0.130	0.145	0.171	0.128	0.185
(SD)	0.074	0.051	0.059	0.085	0.068	0.087	0.094	0.067	0.099
Perimeter Mean (μm)	1.452	1.236	1.332	1.608	1.461	1.446	1.647	1.427	1.669
(SD)	0.413	0.413	0.357	0.494	0.511	0.509	0.544	0.474	0.524
Feret's diameter (μm)	0.374	0.353	0.355	0.505	0.499	0.45	0.429	0.429	0.407
(SD)	0.15	0.148	0.18	0.362	0.377	0.214	0.243	0.256	187
n	362	244	253	243	270	298	265	242	334

Each cohort consisted by three hamsters. SARS-CoV-2: Severe acute respiratory syndrome coronavirus 2. IAV: influenza A virus. SD: Standard deviation.

Supplementary Table S3. Comparisons of morphometric and shape descriptors of intermyofibrillar mitochondria

	Mock			SARS-CoV-2			IAV		
Animal ID	#1	#2	#3	#1	#2	#3	#1	#2	#3
Area Mean (μm)	0.061	0.064	0.057	0.12	0.089	0.089	0.075	0.078	0.076
(SD)	0.045	0.051	0.051	0.160	0.100	0.064	0.063	0.096	0.071
Perimeter Mean (μm)	0.937	0.927	0.894	1.274	1.189	1.133	1.054	1.055	1.026
(SD)	0.337	0.346	0.410	0.890	0.784	0.477	0.523	0.614	0.458
Feret's diameter (μm)	0.526	0.559	0.489	0.641	0.586	0.563	0.792	0.568	0.65
(SD)	0.182	0.182	0.187	0.21	0.228	0.226	0.338	0.22	0.225
n	569	509	723	868	526	488	585	714	477

Each cohort consisted by three hamsters. SARS-CoV-2: Severe acute respiratory syndrome coronavirus 2. IAV: influenza A virus. SD: Standard deviation.

Supplementary Data S1. Summary of Gene Set Enrichment Analysis (GSEA) results. Within this Excel file, each row corresponds to a gene set from the Molecular Signatures Database (MSigDB, version 7.5.1). Columns A-D: gene set annotation, including MSigDB collection and subset (e.g., source pathway database or GO term category), gene set name, gene set size (i.e., number of genes with 1:1 hamster:human homolog relationship in MesAur1.0); columns E-V: Normalized Enrichment Score (NES), nominal p value, and FDR q value for each gene set in each pairwise comparison, as computed by GSEA (version 2.2.1). Rows are sorted in ascending order by columns T, Q and N (i.e., NES for S d60 vs M, S d30 vs M, and S d3 vs M comparisons, in that order) and filtered to show the 415 gene sets with significant coordinate downregulation relative to mock group in SARS-CoV-2-infected animals at both 30- and 60-days post infection (dpi) (*i.e.,* NES < 0 and FDR q < 0.1 for both S d30 vs M and S d60 vs M).
